# The Surgery of the Chest

**Published:** 1897-03

**Authors:** 


**The Surgery of the Chest.** By STEPHEN PAGET. Pp. x., 479. Bristol: John Wright & Co. 1896.

The surgery of the chest seems scarcely to lend itself to the formation of a book in the same way that the surgery of the abdomen does, but we are quite ready to admit with Mr. Paget that "now is the time when such a book can hardly fail to be useful: first, because there is a vast store of good material to be worked into it; next, because there are signs that we have reached a stage, in this portion of our art, beyond which, on our present lines, we cannot advance much further."

The book before us is divided into two parts; the first treating of injuries, and the second of diseases of the chest. The subject of fractures of the ribs is somewhat briefly dealt with, the author referring chiefly to the writings of Malgaigne, Hamilton, and Poland. Injuries to the lung and pleura are treated at greater length, and particular attention is given to the interesting subjects of pneumothorax and surgical emphysema. We could wish, however, that on such subjects as these the author had given us his own opinions and advice, instead of simply presenting us with the varied views of others and then leaving us to take our own choice. His plan has been to give a short outline of what has been written by other surgeons and then to fill in with abstracts of clinical cases illustrating various aspects of the diseases referred to. The result is that the book is very dry and uninteresting. It is hardly full enough for a book of reference, but is well adapted to serve the purpose of any surgeon wishing to hastily refresh himself on any point in which he may be interested at the moment.

In the second part of the book we find the subject of empyema very fairly dealt with. We are glad to see that the author protests—though somewhat mildly—against the present practice of resection of a portion of rib as a routine treatment of empyema. He refers to fifteen cases of empyema treated by one surgeon by incision without resection, every one of them successful, and says that he himself has treated it several times in this way with success; but he remarks that incision without resection is, at present, out of favour, and the removal of an inch of rib has come to be almost an integral part of the operation. We are happy to say that resection of a portion of a rib is by no means a routine practice in Bristol, and we could furnish reports of scores of cases treated successfully by simple incision and drainage.

The operation of resection of large portions of the chest-wall for persistent empyema with fistula is fully described, and Schede's operation of "thorax-resection" and Estlander's operation of removal of portions of several ribs are particularly mentioned.

On the subject of operating for abscess in the lung the author is wisely very cautious. He alludes freely to the difficulties and dangers of the operation, but points to the encouragement afforded by a long record of successful cases. Even in the unfavourable case of operating for a tubercular cavity in the lung, proof is given that a patient may be saved from acute distress during the short span of life which is all that remains to him, and sufficient directions are found in this work to guide the surgeon as to the best methods of performing these and other operations on the lung.

We have no space to allude to the other topics in the book, which we can recommend as the painstaking work of one who has evidently studied the subject with great care and has performed his duty with scholarly ability.

